# ﻿Taxonomic novelties in *Haplopappus* (Asteraceae, Astereae) from Chile

**DOI:** 10.3897/phytokeys.237.114461

**Published:** 2024-01-29

**Authors:** Nicolás García, Arón Cádiz-Véliz, Macarena Villalobos, Vanezza Morales

**Affiliations:** 1 Herbario EIF & Laboratorio de Evolución y Sistemática, Facultad de Ciencias Forestales y de la Conservación de la Naturaleza, Universidad de Chile, Avenida Santa Rosa 11315, La Pintana, Santiago, Chile Universidad de Chile Santiago Chile; 2 Departamento de Botánica, Facultad de Ciencias Naturales y Oceanográficas, Universidad de Concepción, Casilla 160C, 4030000, Concepción, Chile Universidad de Concepción Concepción Chile; 3 Instituto de Ecología y Biodiversidad (IEB), Concepción, Chile Instituto de Ecología y Biodiversidad (IEB) Concepción Chile; 4 Museo Nacional de Historia Natural, Interior Quinta Normal s/n, casilla 787, Santiago, Chile Museo Nacional de Historia Natural Santiago Chile

**Keywords:** Central Chile, Compositae, IUCN, new species, taxonomy

## Abstract

Two new species of *Haplopappus* (Asteraceae) from central Chile are described in this article. *Haplopappuscolliguayensis***sp. nov.** is restricted to La Chapa hill, Colliguay, Valparaíso Region, and is most similar to *H.undulatus* but differs from the latter in its stem indumentum, leaf shape and margin, and synflorescence arrangement. *Haplopappusteillieri***sp. nov**. has been recorded from four high-Andean localities in the Choapa, Petorca, Rocín and Aconcagua river basins, and is most similar to *H.punctatus* but differs from the latter in its leaf length and margin, number of peduncles per twig, width of outer phyllaries, number of ray florets per capitulum, and achene dimensions. Additionally, we propose the reinstatement of *H.kingii* in agreement with an exhaustive revision of type material and protologues, as well as the study of herbarium specimens. *Haplopappuskingii* is restricted to mountainous areas in the southern portion of the Atacama Region, and resembles *H.parvifolius* and *H.retinervius* but differs from both by its leaf margin and apex, besides additional differences from each. We provide morphological descriptions, field images, distributional maps, conservation assessments, and taxonomic notes for the three species treated, as well as illustrations for the novel taxa.

## ﻿Introduction

*Haplopappus* Cassini is a strictly endemic South American genus in the tribe Astereae Cassini (Nesom and Robinson 2007; Funk et al. 2009), composed of 67 specific and intraspecific taxa (Klingenberg 2007; García et al. 2018), distributed in southern South America. Most of the genus diversity is restricted to Chile with 65 taxa, of which 48 (74%) are endemic to this country (Rodríguez et al. 2018).

*Haplopappus* comprises shrubs, subshrubs and only a few herbaceous taxa (Hall 1928; Cabrera 1934, 1971, 1978; Klingenberg 2007; Nesom and Robinson 2007; Tortosa and Bartoli 2002a, b; Bartoli and Tortosa 2013, 2014). Stems, leaves and phyllaries usually bear glandular trichomes giving them a glutinous to villous-glandular aspect. Phyllaries are organized in numerous series (3–6) and are coriaceous, appressed to recurved, tips acute to spiniform, with an herbaceous portion in a distinct apical green patch. True ray florets are usually yellow, in one series when present and disc florets are usually yellow, numerous, hermaphroditic, narrowly funnelform. Pappus bristles are unequal and numerous, arranged in 2–3 series, slightly connate in a basal ring (Gay 1849; Reiche 1902; Hall 1928; Cabrera 1934; Nesom and Robinson 2007; Klingenberg 2007; Tortosa and Bartoli 2002a, 2003; Bartoli and Tortosa 2013, 2014). An exhaustive phylogeny is lacking for this genus, therefore, its current intrageneric classification is based on morphological similarity, dividing *Haplopappus* in three subgenera and five sections (Klingenberg 2007; García et al. 2018).

Recent floristic studies in the Valparaiso Region of Central Chile led to the discovery of two new *Haplopappus* species. In addition, we also propose the reinstatement of *H.kingii* (Phil.) Reiche, a name that was placed under the synonymy of *H.remyanus* Wedd. by Klingenberg (2007).

Herein, we describe *H.colliguayensis* and *H.teillieri*, two new species from Central Chile, and provide information to support the taxonomic status of *H.kingii*. In addition, we provide illustrations and/or photographs, distribution maps, conservation status assessments, and taxonomic notes for all the species treated here.

## ﻿Methods

At first, recently collected material was checked against taxonomic keys and descriptions of *Haplopappus*, which were provided by Gay (1849), Philippi (1858), Reiche (1902), Hall (1928), Brown and Clark (1982) and Klingenberg (2007). Then, the specimens were compared to general and type material held at Chilean herbaria (CONC, EIF, SGO, ULS). Digital images of specimens available on the websites of the following herbaria were also examined: BAA, BM, CAS, E, F, GH, K, LIL, M, MSB, NY, P, PH, S, SI, US, WU. Botanical terminology of the descriptions follows Beentje (2012). Leaf widths were measured over the widest portion of the lamina not considering the teeth, which were described separately. Two capitula per sample were rehydrated in 70% ethanol for 24 hours and were subsequently dissected. The different parts of the capitula (e.g., phyllaries, florets, gynoecium) were mounted on a white cardboard and scanned; measurements of capitulum parts were obtained with the free software ImageJ (Schneider et al. 2012). Distribution maps and the estimation of the area of occupancy and the extent of occurrence (*sensu*IUCN 2022) were generated using the GIS software ArcGis version 10.4 (ESRI 2015).

## ﻿Taxonomic treatment

### ﻿ Haplopappus colliguayensis

Taxon classificationPlantaeAsteralesAsteraceae

M.A.Villalobos, V.Morales & Nic.García sp. nov.

5118D427-B108-56BE-BED7-CD1A360BCEDB

urn:lsid:ipni.org:names:77335209-1

Figs 2 , 3

#### Diagnosis.

*Haplopappuscolliguayensis* is similar to *H.undulatus* Klingenb., but differs from the latter by its stems with capitate glands and multicellular flagelliform trichomes (vs. sessile glands), leaves oblong to lanceolate (vs. obovate to oblanceolate), leaf margins flat, entire to shortly dentate with up to 5 teeth per side (vs. margins undulate, conspicuously dentate to serrate with 5–9 teeth per side), and paniculiform synflorescences with two or more capitula (vs. solitary capitulum).

#### Type.

Chile. Región de Valparaíso: Provincia de Marga Marga, Comuna de Quilpué, Colliguay, cerro La Chapa, 957 m a.s.l., 33°9'13"S, 71°7'54"W, 27 February 2023, *N. García, M.A. Villalobos, V. Morales, A. Cádiz-Véliz, S. Olfos & O. Ovalle 6783* (holotype: EIF 17304!; isotypes: CONC!, JBN!, SGO!, SI!, VALPL!).

#### Description.

Shrubs 0.4–0.8 m high, 0.1–0.3 m in diameter, ascending to erect, aromatic. Branching at base, some branches dry or with dry leaves and leaf scars on proximal half, leaves green towards the middle and distal portion of branches. Stems slightly corrugated, coppery, densely covered by minute capitate glandular trichomes and scattered multicellular flagelliform (2.0 mm) trichomes (same indumentum up to the peduncles). Leaves (0.5–)1.5–2.5(–4.0) × (0.1–)0.3–0.6(–1.0) cm; laminae oblong to lanceolate, gradually reduced in size towards the tip of branches, usually around five times as long as wide; bases cordate to obtuse; apices acute to acuminate, mucronate; margins entire to irregularly dentate, teeth 0.2–2 × 0.5–1.0 mm at the base, mucronate, (0–)1–3(–5) teeth on each side; leaves densely covered by short capitate glandular trichomes, multicellular, 0.25 mm long, and scattered subulate eglandular trichomes and flagelliform trichomes, membranous-papery to coriaceous, simple, sessile, alternate; venation with a dominant midvein and 2–4 basal secondary veins running more or less parallel to it, forming an arch with upper secondaries, overall reticulate due to tertiary veins. Capitula radiate, heterogamous, usually the apices of the main axis and (1–)2–5(–8) short secondary ramifications carry a single capitulum each, less frequently solitary on the main axis or up to 2 capitula per secondary branch, within a paniculiform synflorescence (paniculodium *sensu*Klingenberg 2007), that tends to take the aspect of a pseudoraceme. Peduncles up to 6 mm long or obsolete due to leaves growing up to the capitulum. Involucres 8–11 × 6–10 mm, campanulate to cylindrical. Phyllaries spirally arranged in 6–7 series, covered by short capitate glandular trichomes, border erose, mucronate; external series 2.0–2.5 × 0.5 mm at its widest, linear, mostly green (parenchymatous), sclerenchymatous basally, distal half to two thirds reflexed 30–45°, acute; intermediate series 4–6 × 0.6 mm at its widest, linear, green and reflexed 30–45° on distal half, acute, sclerenchymatous on proximal half; internal series 6.5–7 × 0.9 mm across the middle area, linear, apices straight or shortly reflexed, acute, parenchymatous on distal third, sclerenchymatous elsewhere, margins and below hyaline. Receptacles flat, epaleate, alveolate. True ray florets with tubular portion 4.0 × 0.3 mm and limbs 7.5 × 2.7 mm at its widest, with 4(–6) marked veins, both lateral bifurcating around the middle portion, ending in 2–3 small apical teeth, glabrous, 13–17 per capitulum, pistillate, corollas saffron (yellow-orange), zygomorphic, tubular portion covered with short, multicellular eglandular trichomes, becoming denser towards the throat; styles 4 mm long, divided asymmetrically into two branches; ovaries 1.5 × 0.5 mm, densely strigose. Disk florets 6.5–8.0 × 1.5–1.8 mm at their widest portion, numerous (60–70), perfect, corollas saffron, actinomorphic, infundibuliform, with 5 short lobes, 0.6–0.9 mm deep, edges thickened, shortly papillate; anthers 2.5 mm long; styles 4 mm long, with two style branches, 2.5 mm long each, papillate border on proximal half, differentiated distal half with multicellular collecting trichomes, apical triangular portion papillate; ovaries 1.5–2.5 × ~0.8 mm, densely strigose. Cypselae: achenes 4.0–4.5 × 0.8–1.2 mm, oblong-lanceolate, slightly flattened, asymmetrical, ribbed, only two lateral ribs prominent, covered by white, multicellular, stiff trichomes, 0.5–1 mm long, appressed or slanted up to 45° relative to the exocarp; pappus 3.0–5.5 mm long in ray florets and 3.0–6.0 mm long in disk florets, with numerous bristles (ca. 44), white, persistent, barbellate.

#### Distribution and habitat.

**Figure 1.**
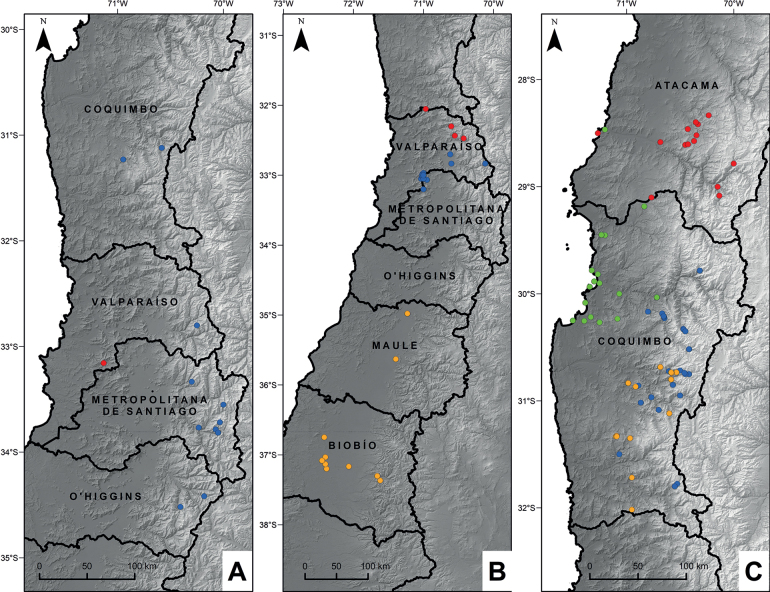
Distribution of *Haplopappus* in central Chile **A***H.colliguayensis* (red dot), *H.undulatus* (blue dots) **B***H.teillieri* (red dots), *H.integerrimus* (blue dots), *H.punctatus* (orange dots) **C***H.kingii* (red dots), *H.parvifolius* (green dots), *H.remyanus* (blue dots), *H.retinervius* (orange dots).

*Haplopappuscolliguayensis* has been recorded only in the La Chapa hill, Colliguay (~33.1°S; Fig. 1A), which is part of the coastal mountain range (*cordillera de la Costa*) between the Aconcagua and Maipo rivers. It inhabits rocky outcrops in south- to southwest-facing positions from the base of the hill (680 m a.s.l.) towards its summit (~1680 m a.s.l.). The surrounding zonal vegetation corresponds to sclerophyllous coastal forest; however, the vegetation associated with the rocky outcrops corresponds to a xerophilous scrub with predominance of *Adesmiapirionii* I.M.Johnst., *Gochnatiafoliolosa* (D.Don) D.Don ex Hook. & Arn., *Puyacoerulea* Lindl., and *Chusqueacumingii* Nees.

#### Etymology.

The specific epithet refers to Colliguay, a locality situated to the south of the city of Quilpué in the Valparaíso Region of Chile.

**Figure 2.**
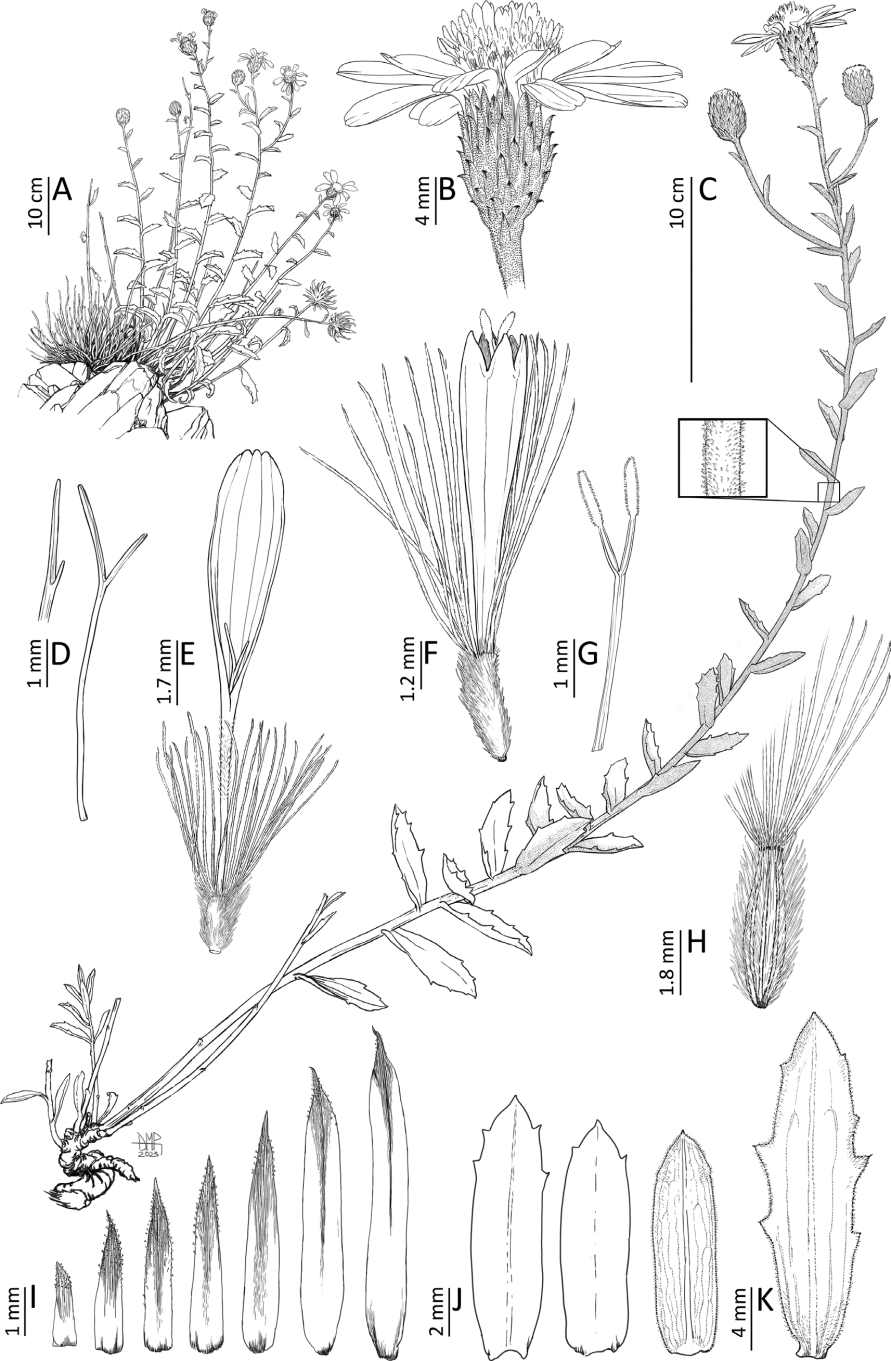
*Haplopappuscolliguayensis* M.A.Villalobos, V.Morales & Nic.García **A** habit **B** capitulum **C** flowering branch, inset shows glandular pubescence on stem **D** style and asymmetrical stigmatic branches of true ray florets **E** true ray floret **F** disk floret **G** style and stigmatic branches of disk floret **H** achene **I** series of phyllaries **J** leaves (“bracts”) subtending capitula **K** leaf. Drawn by Daniel Martinez Piña from *N. García et al. 6783*, 6785.

#### Phenology.

This species has been recorded flowering in February, but the period probably extends between January and March. Fruits have been recorded between February and April.

**Figure 3.**
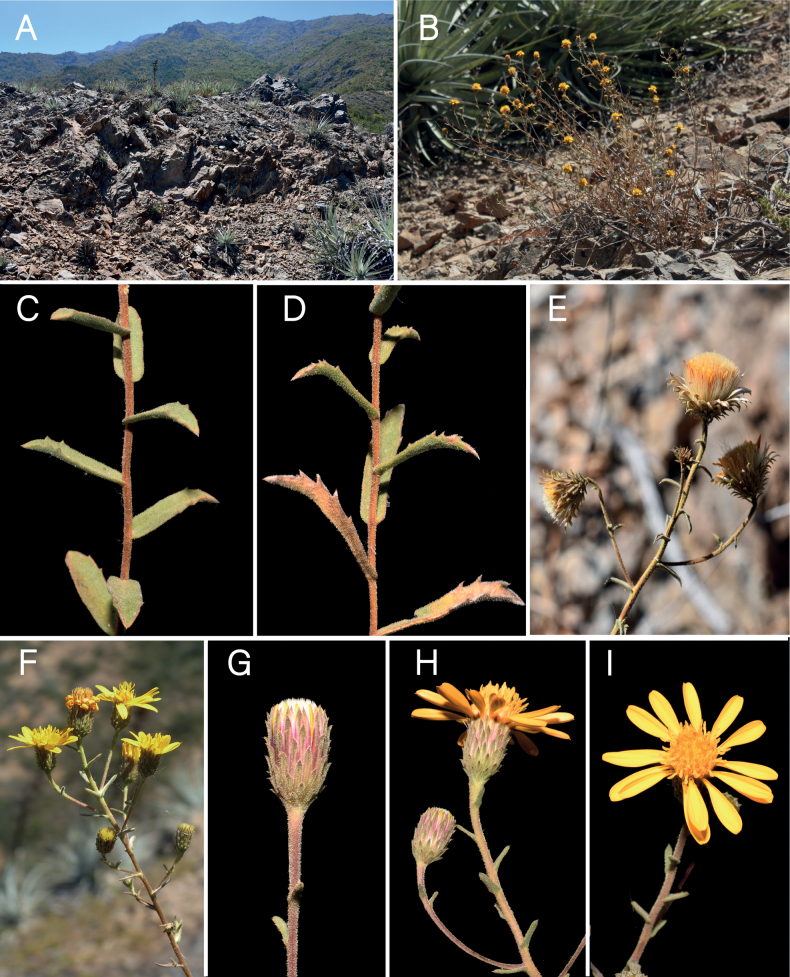
*Haplopappuscolliguayensis* M.A.Villalobos, V.Morales & Nic.García **A** habitat **B** habit **C, D** detail of stem and leaves **E** fruiting capitula **F** flowering branch showing paniculiform synflorescence **G** immature capitulum **H** capitulum, side view **I** capitulum, top view. Photographs by Arón Cádiz-Véliz (**A, C–E, G–I**), Macarena Villalobos (**B**), Nicolás García (**F**).

#### Conservation status.

According to IUCN (2022), *Haplopappuscolliguayensis* can be considered as Critically Endangered (CR) according to criteria B2ab(iii, v), because its Area of Occupancy (AOO) is <10 km^2^ (4 km^2^). The criterion “a” was selected because it is known from a single locality. Although it is estimated that the only known population maintains around 1,000 individuals, all of them grow concentrated in a reduced area, which is susceptible to be affected by natural catastrophes or anthropogenic interventions (e.g., fires, droughts). The criterion “b(iii)” was selected because there is an inferred and projected decline in the extent and the quality of the habitat, given projected replacement of the vegetation due to climate change and the high probability of fire occurrence in the area where the species inhabits. According to MMA (2023b), the climate scenarios for 2050 predict an increase in temperature (of 1–2 °C) and a decrease in precipitation (~60–80 mm) in the county of Quilpué, where the species has been recorded. The predicted changes in precipitation allowed to classify this area under high risk of loss of flora (MMA 2023a). Moreover, the region of Valparaíso is one of the Chilean regions that has experienced one of the highest numbers of fires and its burned area was extensive between 1985–2018 (González et al. 2020). As a proof of this risk, a major fire that consumed 10 km^2^ in December of 2022, affected a valley contiguous to Colliguay and reached only 2 km away from the location of *H.colliguayensis*. The persistence of this trend with the probability of such future events in La Chapa hill would generate a decrease in the number of mature individuals, affecting the persistence of the species (criterion “b(v)”).

#### Additional specimens examined

**(paratypes).** Chile. Región de Valparaíso: Provincia de Marga-Marga, Comuna de Quilpué, Colliguay, cerro La Chapa, 680 m a.s.l., 17 April 2019, *N. García, M.A. Villalobos & N. Godoy 5561* (EIF); 679 m a.s.l., 15 October 2019, *M.A. Villalobos & J.P. Madriaga 111* (EIF, SGO); 1047 m a.s.l., 10 December 2019, *M.A. Villalobos, V. Farías & P. Villalobos 154* (EIF, SGO, CONC); 1675 m a.s.l., 27 December 2019, *N. García, M.A. Villalobos & V. Villablanca 5720* (EIF); 735 m a.s.l., 27 February 2023, *N. García, M.A. Villalobos, V. Morales, A. Cádiz-Véliz, S. Olfos, O. Ovalle 6785* (EIF, SGO).

#### Taxonomic notes.

Given its phyllaries acute, bracts up to the capitula similar to the cauline leaves, and peduncles not distinctly different from the twigs, *H.colliguayensis* can be assigned to H.sect.Grindelioidae Klingenb. (Klingenberg 2007). The new species most closely resembles *H.undulatus*, but differs in characters mentioned in the diagnosis and in its distribution restricted to xerophilous/rupiculous scrub in the coastal range, in contrast to the latter which is found in high-montane (i.e., alpine) scrub in the main Andes mountain range between the Valparaíso and O’Higgins regions (Fig. 1A). *Haplopappuscolliguayensis* can also be compared to *H.grindelioides* (Less.) DC., which besides having a much southern distribution between the Maule and Los Ríos regions (vs. Valparaíso Region), also has solitary capitula (vs. paniculiform synflorescence with two or more capitula), stems tomentose (vs. glandulose), and outer series of phyllaries 1.7–2 mm wide (vs. 0.5 mm wide) (Klingenberg 2007).

### ﻿ Haplopappus teillieri

Taxon classificationPlantaeAsteralesAsteraceae

A.Cádiz-Véliz, V.Morales & Nic.García sp. nov.

DAD2C512-1B3F-52B4-A9E4-A05AF596DD83

urn:lsid:ipni.org:names:77335471-1

Figs 4 , 5

#### Diagnosis.

*Haplopappusteillieri* is similar to *H.punctatus* (Willd.) H.M.Hall, but differs from the latter in its leaves 1–2.5 cm long (vs. 3–6 cm), leaf margin always entire (vs. entire to up to 6 teeth per side), 1–3 peduncles per twig (vs. 2–6 peduncles per twig), outer series of phyllaries 1.2–1.8 mm wide (vs. 0.5–1.0 mm), ~7 ray florets per capitulum (vs. 10–12), and achenes 6.5–7 × 0.8–1.0 mm (vs. 3–4.4 × 1.5–1.8 mm).

**Figure 4.**
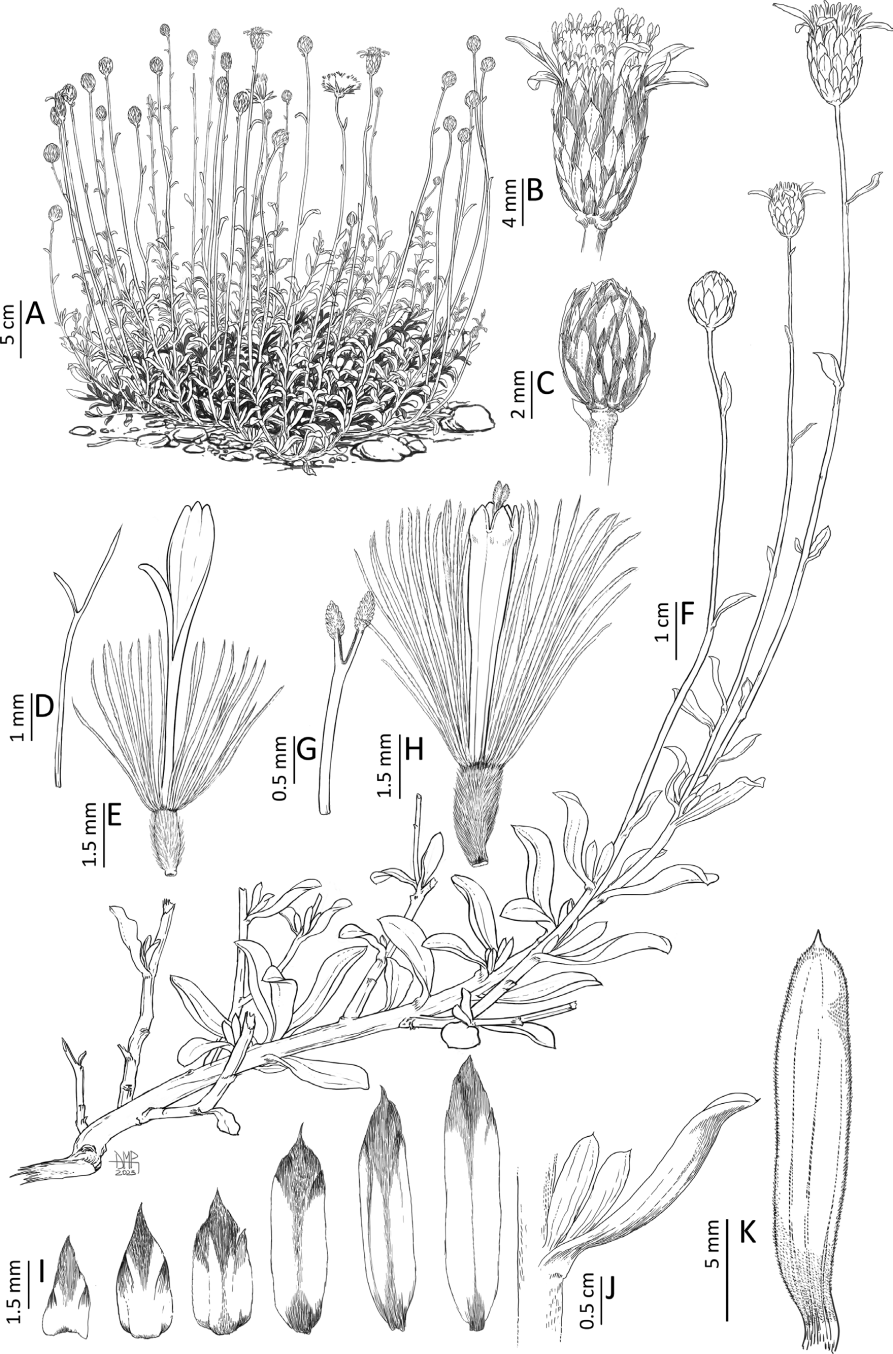
*Haplopappusteillieri* A.Cádiz-Véliz, V.Morales & Nic.García **A** habit **B** capitulum **C** immature capitulum **D** style and asymmetrical stigmatic branches of true ray floret **E** true ray floret **F** flowering branch **G** style and stigmatic branches of disk floret **H** disk floret **I** series of phyllaries **J** fascicle of leaves **K** leaf. Drawn by Daniel Martinez Piña from *A. Cádiz-Véliz et al. 991*.

#### Type.

Chile. Región de Valparaíso: Provincia de San Felipe de Aconcagua, Comuna de Putaendo, río Rocín, sector Las Tejas, 2530 m a.s.l., 32°28'22"S, 70°25'25"W, 04 January 2023, *A. Cádiz-Véliz, J.L. Álvarez & S. Grau 991* (holotype: EIF 17305!; isotypes: CONC!, JBN!, MO!, SGO!, SI!, VALPL!).

**Figure 5.**
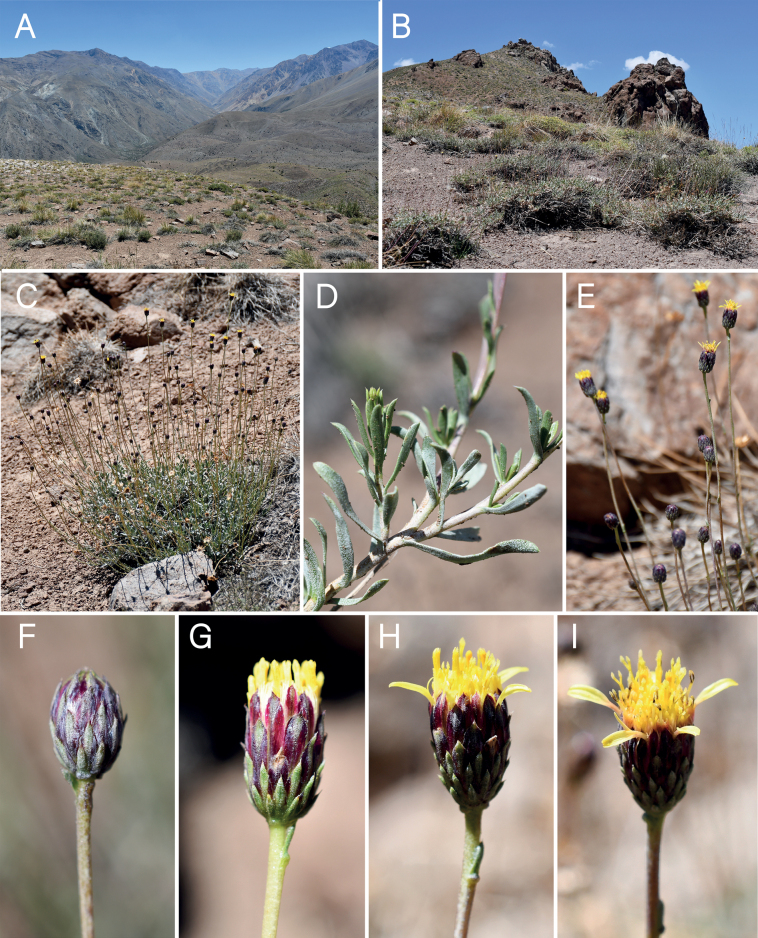
*Haplopappusteillieri* A.Cádiz-Véliz, V.Morales & Nic.García **A** general view of the Rocín valley **B** habitat in Andean scrub-grassland of *Chuquiragaoppositifolia, Festuca acanthophylla* and *H.teillieri***C** habit **D** detail of branches and leaves **E** flowering branches **F** immature capitulum **G** homogamous mature capitulum **H, I** heterogamous mature capitula. All photographs by Arón Cádiz-Véliz.

#### Description.

Shrubs 0.25–0.4(–0.5) m high and 0.35–0.7 m in diameter, ascending to erect, with slight citric odor. Stems growing parallel to the ground at first and branching later in an ascending way. Stems furrowed, papery bark at base, greenish near the base, turning yellowish to reddish towards the apex, sparsely covered by sessile glands (same indumentum up to the peduncles), glutinous. Leaves (0.5–)1.0–2.5(–3.0) × (0.1–)0.2–0.3(–0.55) cm; lamina narrowly lanceolate to oblanceolate, rarely linear, gradually reduced in size towards the tip of branches, around five to eight times as long as they are wide; bases attenuate; apices acute to acuminate, ending in a slender seta, 0.2–0,5 mm long; margins entire, scabrid due to short subulate trichomes; leaves glutinous, covered by sessile glands throughout and sparsely by short subulate unicellular trichomes (~0.25 mm long) on margins and upper central vein, coriaceous, simple, sessile, alternate or frequently fasciculate with 3–5 smaller leaves on the axils of major leaves; venation reticulate and inconspicuous. Capitula radiate and heterogamous, rarely discoid and homogamous, with 3–4 small subulate leaf-like bracts at the base, solitary on the apex of a long peduncle. Peduncles up to 28 cm long, 0.6–1.0 mm wide at base, 1–3 per twig, with 3–8 evenly distributed subulate bracts. Involucres 9–14 × 8–12 mm, cylindrical. Phyllaries spirally arranged in 5–6 series, covered by sessile and short capitate glands, glutinous, ending in a slender seta; external series 2.7–4.7 × 1.2–1.8 mm at its widest, ovate to elliptic, mostly green (parenchymatous), sclerenchymatous basally, margin purplish along the middle and towards the tip, straight, acuminate; intermediate series 4.4–7.4 × 1.6–2.0 mm at its widest, lanceolate to oblong, green-purplish on distal half, sclerenchymatous on proximal half, straight or only tip reflexed, acuminate; internal series 7.4–8.3 × 1.5–2.0 mm across the middle area, ligulate, apices straight or shortly reflexed, acuminate, parenchymatous and purplish on distal third, sclerenchymatous elsewhere, margins and below hyaline. Receptacles flat, epaleate, alveolate. True ray florets with tubular portion 4.7–5.5 × ~0.4 mm and limbs 2.3–3.0 × ~1.0 mm at its widest, with 3–4 marked veins ending in 3 small apical teeth, also 2–3 segments fused forming the limb and an extra shorter segment free, glabrous, ~7 per capitulum, pistillate, corollas pale saffron, zygomorphic, tubular portion covered with short, multicellular eglandular trichomes; styles 4 mm long, ending in two asymmetrical style branches 1.2 and 0.7 mm long each or undivided, glabrous; ovaries 1.0–1.5 × ~0.8 mm, densely strigose. Disk florets 5.6–6.6 × 0.7–0.8 mm at their widest portion, numerous (~40), perfect, corollas pale saffron, actinomorphic, infundibuliform, with 5 short lobes, 0.3–0.8 mm deep, edges thickened, shortly papillate; anthers ~2.5 mm long; styles 5.3 mm long, with two style branches, 1.2–1.3 mm long each, apical triangular portion papillate; ovaries 1.0–1.5 × ~0.5 mm, densely strigose. Cypselae: achenes 6.5–7.0 × 0.8–1.0 mm, linear-oblanceolate, slightly flattened, asymmetrical, ribbed, only two lateral ribs prominent, densely covered by white, stiff trichomes, ~0.25 mm long, slanted 20–45° relative to the exocarp; pappus 4.5–5.5 mm long in ray florets and 3.1–7.0 mm long. in disk florets, with numerous bristles (57–58), ochreous, persistent, barbellate.

#### Distribution and habitat.

*Haplopappusteillieri* inhabits the Mediterranean Andean low scrub of *Chuquiragaoppositifolia* D.Don and *Nardophyllumlanatum* (Meyen) Cabrera, between 1500–2600 m a.s.l. (Luebert and Pliscoff 2017). Only four populations have been recorded in the Andes, along the valleys of rivers Choapa, Petorca, Rocín and Aconcagua (Fig. 1B). It seems to be a very rare species but it can become locally dominant.

#### Etymology.

The specific epithet *teillieri* honours the Chilean botanist Sebastián Teillier Arredondo (1956–), who has made significant contributions to the knowledge of the vascular flora of Chile.

#### Phenology.

*Haplopappusteillieri* starts flowering in January, probably extending its bloom until early March. Fruits from February onwards.

#### Conservation status.

*Haplopappusteillieri* was rarely collected since 1924, within a very restricted area in the Andes mountains of the Choapa and San Felipe de Aconcagua provinces (Fig. 1B), which suggests that it is a rare species. Here, we propose the species conservation status as Endangered (EN), considering the criteria B1+B2ab(iii). It has been assessed under the criterion “B1” as its Extent of Occurrence (EOO) is <5,000 km^2^ (266 km^2^), while the criterion “B2” corresponds to the Area of Occupancy (AOO) <500 km^2^ (16 km^2^). Criterion “a” is invoked given the species presence in less than five localities (4). Its populations are potentially threatened since all these mountainous areas are located within a zone of high interest for mining development and consequently is fully covered by mining petitions (SONAMI 2023). The criterion “b(iii)” corresponds to the inferred and projected decrease in the quality of the habitat due to the presence of bovine and caprine livestock and mining activities (e.g., opening of roads, prospecting, excavations, removal of soil and vegetation due to installation of facilities). The habitat will also be affected by climate change the consequences of which are a decline in precipitation (35–50 mm) and temperature increase (~2 °C) (MMA 2023a). In this sense, the species inhabits an area that is projected to suffer a moderate to high risk of loss of the flora because of precipitation decrease (MMA 2023a).

#### Additional specimens examined

**(paratypes).** Chile. Región de Coquimbo: Provincia de Choapa, Comuna de Salamanca, Cuenca Camisas, sector Antena, 2350 m a.s.l., 32°3'S, 70°58'W, 02 July 2000, *G. Arancio & F. Squeo 13236* (ULS 13692, 13693). Región de Valparaíso: Provincia de Petorca, valle del río Alicahue, 1500 m a.s.l., 32°17'56"S, 70°36'10"W, March 2016, *S. Teillier & J. Torres-Mura 8039* (CONC 182727); Provincia de San Felipe de Aconcagua, Laguna del Copín, 3000 m a.s.l., 32°26'S, 70°33'W, April 1924, *C. Joseph 15052* (CONC 59861).

#### Taxonomic notes.

Considering its phyllaries acute ending in a slender seta, bracts subulate, peduncles distinct from the twigs, leaves mostly narrowly lanceolate to oblanceolate, and sterile ray florets, *H.teillieri* clearly belongs in H.sect.Gymnocoma Nutt. (Brown and Clark 1982; Klingenberg 2007). Due to its capitula with ray florets and tips of the phyllaries straight to slightly spreading, it most closely resembles *H.punctatus*, with which it is directly contrasted in the diagnosis. It also resembles *H.integerrimus* (Hook. & Arn.) H.M.Hall due to its leaves glutinous and scabrid, presence of 1–3 peduncles per twig, and ray florets inconspicuous (~7–9 mm long), but *H.teillieri* differs from the latter species by its shorter and narrower leaves ((0.5–)1.0–2.5(–3.0) × (0.1–)0.2–0.3(–0.55) cm vs. 3.0–7.5 × 0.3–0.9 cm), leaf margin always entire (vs. 0–6 teeth per side), peduncles 0.6–1.0 mm wide at base (vs. 1.5–3.0 mm), tips of phyllaries straight to slightly spreading (vs. spreading to recurved), and internal series of phyllaries 1.5–2.0 mm wide (vs. 1.2–1.5 mm) (Klingenberg 2007).

### ﻿ Haplopappus kingii

Taxon classificationPlantaeAsteralesAsteraceae

(Phil.) Reiche, Anales Univ. Chile 109: 41. 1901, as “ kingi”

B85F8311-F155-523F-A104-132B8A4B20D7

Figs 6 , 7

Haplodiscus kingii Phil., Anales Univ. Chile 87: 615. 1894, as “*kingi*”.

#### Type.

Chile. Región de Atacama: Provincia de Huasco, Carrizal, 1885, *T. King 62* (holotype: SGO! [SGO000005614]; isotype: E! [E00253112]).

**Figure 6.**
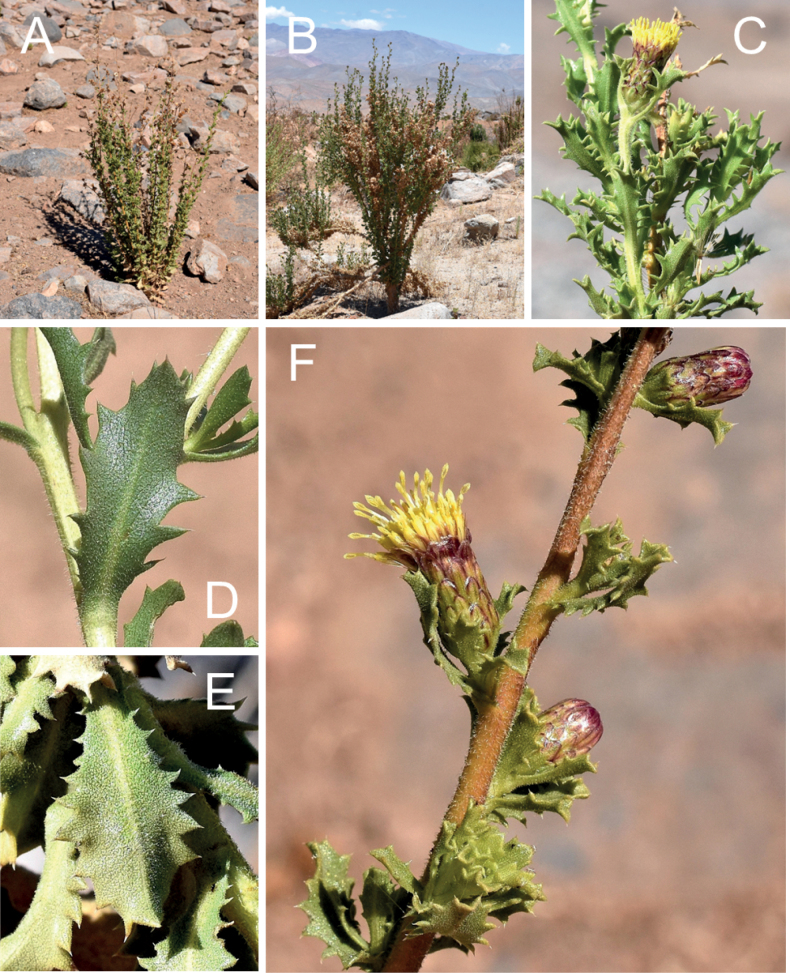
*Haplopappuskingii* (Phil.) Reiche **A, B** habit **C** flowering branch **D, E** detail of the abaxial and adaxial face of leaves, respectively, showing the characteristic hispid pubescence **F** branch showing several sessile capitula, fascicles of leaves and hispid stem. All photographs by Philippe Dandois.

#### Description.

Shrubs, ascending to erect, aromatic. Branching unknown. Stems slightly corrugated, coppery to yellowish, sparsely covered by minute stiff and bulbous trichomes (<0.5 mm) and scattered sessile glands (same indumentum up to the peduncles), glutinous. Leaves (1.0–)1.5–2.6(–3.1) × (0.3–)0.4–1.2(–1.9) cm; laminas oblong to oblanceolate, gradually reduced in size towards the tip of branches, usually around twice as long as they are wide; bases truncate to shortly decurrent; apices acute, rarely obtuse, mucronate; margins strongly dentate, teeth 2.0–3.0 × 1.5–3.0 mm at the base, mucronate, (4–)6–8(–9) teeth on each side; leaves densely covered by minute stiff trichomes, multicellular, 0.2 mm long, and sessile yellowish glands, coriaceous, glutinous, simple, sessile, alternate; venation with a dominant midvein and inconspicuous secondary veins. Capitula discoid, homogamous, usually clustered on short secondary branches, up to 3 capitula per branch, within a paniculiform synflorescence (paniculodium sensu Klingenberg 2007), that tends to take the aspect of a pseudoraceme. Peduncles obsolete due to leaves growing up to the capitulum, rarely up to 3 mm long. Involucres 10–12 × 9–18 mm, cylindrical. Phyllaries spirally arranged in 7–8 series, hyaline margin from the base to the apex, up to 0.5 mm wide on its widest portion, parenchymatous portion covered by minute sessile glands, mucronate; external series 4.2–5.7 × 1.7–2.5 mm at its widest, oblong to obovate, mostly burgundy (parenchymatous), sclerenchymatous basally, acute; intermediate series 5.4–7.6 × 1.8–2.3 mm at its widest, oblong to oblanceolate, acute, sclerenchymatous on proximal half; internal series 7.7–11.0 × 1.3–2.2 mm across the middle area, linear, apices acute, parenchymatous on distal third, sclerenchymatous elsewhere, margins and below hyaline. Receptacles flat, epaleate, alveolate. Disk florets 6.4–7.0 × 0.6–1.0 mm at their widest portion, numerous (38–61), perfect, corollas pale yellow (on herbarium specimens), actinomorphic, infundibuliform, with 5 short lobes, 0.5–0.8 mm deep, edges thickened, glabrous, rarely shortly papillate; anthers 2.9–3.1 mm long; styles 5.4–6.3 mm long, with two style branches, 1.1–1.9 mm long each, papillate on proximal half, differentiated distal half with multicellular trichomes; ovaries 2.5–3.3 × 0.3–0.7 mm, sparsely hispid. Cypselae: achenes 5.0–6.5 × 2.0 mm, linear, wider towards the middle portion, slightly flattened, asymmetrical, ribbed, only two lateral ribs prominent, hispid; pappus 4.1–9.5 mm long, with numerous bristles (55–61), chestnut to cinnamon, persistent, barbellate.

**Figure 7.**
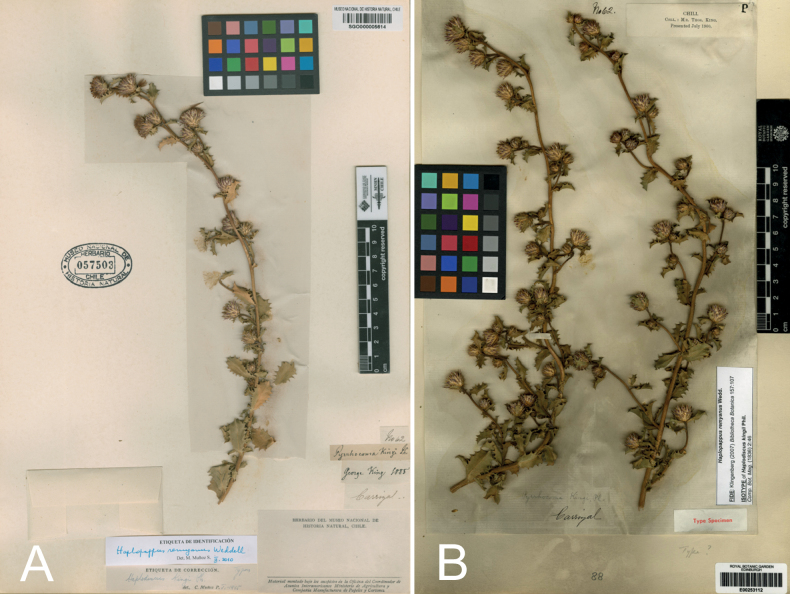
Type specimens of *Haplopappuskingii* (Phil.) Reiche **A** SGO000005614 (holotype) **B** E00253112 (isotype).

#### Distribution and habitat.

This species is endemic to the Atacama Region in Chile (28°25’–29°05'S), mostly occurring in the middle portions of the Carrizal and Huasco river basins (Fig. 1C). It has been registered growing mostly on roadsides in mountainous areas, between 1500–3200 m a.s.l. There is a single record in the coast south of Huasco, which we consider may be an accidental occurrence.

#### Etymology.

The specific epithet honours Thomas King, English citizen who collected several specimens in the Atacama Desert during the late 19^th^ century.

#### Phenology.

Flowering from November to January and fruiting from January to March.

#### Conservation status.

*Haplopappuskingii* is only known from few herbarium specimens and field photographs (P. Dandois, personal communication, 7 July 2023). In accordance with the IUCN (2022), the species is known from 12 localities (Fig. 1C), presenting an estimated Extent of Occurrence (EOO) of 7,087 km^2^ and Area of Occupancy (AOO) of 56 km^2^. Although the estimations of EOO and AOO reach the values of threatened categories (Vulnerable and Endangered, respectively), there is not much information about the current state of the populations. The lack of these data does not allow us to classify the species under any threatened category. However, it is known that the species inhabits an area affected by the development of mining activities (SONAMI 2023) and the severe drought in Central Chile, which has produced a shortfall on normal precipitation of about 20–40% between 2010–2014 in the study area (CR2 2015). Moreover, the projections of climate change to 2050 estimate a decrease in precipitation (2–8 mm) and increase in temperature (1.8–2.5 °C) (MMA 2023b). Consequently, the area where the species is distributed will face a moderate to high risk of the loss of flora given the changes in precipitation (MMA 2023a). Considering all of the above, we inferred a change in the quality of the habitat of *H.kingii* but as the number of known localities exceeds thresholds for threatened categories, we propose the species conservation status as Near Threatened (NT).

#### Additional specimens examined.

**Chile. Región de Atacama**: Provincia de Huasco, El Bronce – Mantos Verdes, 1550 m a.s.l., 28°25'S, 70°21'W, November 2007, *S. Teillier & J. Delaunoy 5566* (CONC 166856); Quebrada Jilguero, crece en quebradas y laderas, abundante, 600 m a.s.l., 28°35'S, 70°41'W, 2 December 2008, *J. Reyes 4802* (ULS); Quebrada La Escoria, precordillera, 28°27'40.93"S, 70°25'39.22"W, 14 March 2013, *G. Mieres s.n.* (CONC 179797); Quebrada El Molle, precordillera, 28°31'7.48"S, 70°20'47.86"W, 11 January 2013, *G. Mieres s.n.* (CONC 179877, EIF 17306); Quebrada La Gloria, precordillera, 28°34'24.38"S, 70°22'7.48"W, 23 August 2012, *G. Mieres s.n.* (CONC 179800); Valle del río Laguna Grande, 2100–3000 m a.s.l., 28°49'S, 70°00'W, 14 February 1981, *M.T. Kalin-Arroyo 81565* (CONC 53397); Río Chollay, crece en ladera y lecho de quebrada, escasa, 2050 m a.s.l., 29°05'S, 70°08'W, 17 January 1994, *G. Arancio et al. 94119* (ULS); Huasco, crece entre rocas, 20 m a.s.l., 28°30'S, 71°16'W, 13 December 2008, *J. Reyes 6260* (ULS).

#### Taxonomic notes.

*Haplopappuskingii* had been considered a distinct species in treatments of *Haplopappus* by Reiche (1902) and Hall (1928). However, Klingenberg (2007) reduced *H.kingii* into the synonymy of *H.remyanus* in H.sect.Leiachaenium DC., a decision that was followed by the latest catalogue of the vascular flora of Chile (Rodríguez et al. 2018). *Haplopappuskingii* can be differentiated from the latter species by its hispid indumentum (vs. glabrous and glutinous), leaves evenly distributed throughout the stem up to the synflorescence (vs. leaves distinctly clustered towards the base of the plant, flowering branches sparsely foliate below the capitula), and outer series of phyllaries 1.7–2.5 mm wide (vs. 2.5–3.0 mm wide).

However, a close inspection of descriptions and herbarium specimens suggest that *H.kingii* better fits within Klingenberg’s (2007)H.sect.Chromochaeta DC., where it most closely resembles *H.parvifolius* (DC.) A. Gray and *H.retinervius* (Kuntze) Klingenb. *Haplopappuskingii* differs from both species by its leaves with mostly flat margin (vs. margin undulate) and leaf apex acute (vs. obtuse to rounded). More specifically, *H.kingii* differs from *H.parvifolius* by its hispid indumentum (vs. glabrous plants), villous achenes (vs. glabrous achenes) and green leaves (vs. glaucous leaves), and from *H.retinervius* by its leaves oblong to oblanceolate (vs. broadly obovate to nearly orbicular) and more than 35 florets (vs. less than 30 florets) per capitulum (Klingenberg 2007).

In the citation of the type material, we recognize Thomas King as the collector of the sample, which differs from the name mentioned on the protologue, “Georgius King” (Philippi 1894: 615). The holotype at SGO (Fig. 7A) is accompanied by four pieces of paper, each of them with the following information: name of the species (“*Pyrrhocomakingii* Ph.”), the name of the collector and the year of collection (“George King 1885”), the locality (“Carrizal”) and what we interpreted as the collection number (“No 62”). On the other hand, the specimen at E (Fig. 7B) is attached to a piece of paper in which it is possible to read “No 62” and “Carrizal”. Additionally, there is a printed label saying “Chili. Coll.: Mr. Thos. King. Presented July 1900.”, that suggests the sample was sent to Edinburgh by Thomas King himself. The labels with the name of the locality and the collection number seem to have the same handwriting, which suggests they were written by the collector. Thomas (or Tomas) King is a well-known collaborator of R.A. Philippi, who sent him several samples of plants from the Carrizal valley in the Atacama Region and some of these samples were used to describe new species (Philippi 1892; e.g. *Leucocorynenarcissoides* Phil., *Alstroemeriakingii* Phil., *Valerianasenecioides* Phil.). Apart from the description of this species, the name Georgius King has not appeared in other publications. Therefore, we assume that Philippi made a mistake when writing the name of the collector on the label of the holotype specimen of *Haplodiscuskingii* at SGO and in the species protologue.

## Supplementary Material

XML Treatment for Haplopappus colliguayensis

XML Treatment for Haplopappus teillieri

XML Treatment for Haplopappus kingii

## ﻿Additional information

### Author ORCIDs

Nicolás García https://orcid.org/0000-0001-9003-1510

Arón Cádiz-Véliz https://orcid.org/0000-0002-7548-1764

Macarena Villalobos https://orcid.org/0000-0001-8897-3429

Vanezza Morales https://orcid.org/0000-0003-3723-8752

### Data availability

All of the data that support the findings of this study are available in the main text or upon direct request to the authors.
